# Fascia Lata Allograft Medial Patellofemoral Ligament Reconstruction—Restore the Nature as Close as Possible

**DOI:** 10.1016/j.eats.2024.103194

**Published:** 2024-08-13

**Authors:** Marcin Mostowy, Michalina Bawor, Krzysztof Bujak, Przemysław A. Pękala, Robert F. LaPrade, Konrad Malinowski

**Affiliations:** a Orthopedic and Trauma Department, Veterans Memorial Teaching Hospital in Lodz, Medical University of Lodz, Lodz, Poland; b Artromedical Orthopedic Clinic, Bełchatów, Poland; c Medical University of Lodz, Lodz, Poland; d Department of Anatomy, Jagiellonian University Medical College, International Evidence-Based Anatomy Working Group, Kraków, Poland; e Faculty of Medicine and Health Sciences, Andrzej Frycz Modrzewski Kraków University, Kraków, Poland; f Lesser Poland Orthopedic and Rehabilitation Hospital, Kraków, Poland; g Twin Cities Orthopedics, Edina, Minnesota, U.S.A.

## Abstract

Various surgical techniques for medial patellofemoral ligament (MPFL) reconstruction have been described, commonly using hamstring auto- or allografts. Despite their widespread use, these techniques have limitations due to the tubular structure of the hamstring tendons, which does not match the flat, sail-like anatomy of the MPFL. Furthermore, over- or undertensioning of the graft due to tunnel misplacement remains a significant risk, even if anatomic and radiologic landmarks are used. To address these issues, a fascia lata allograft MPFL reconstruction with assessment of angular anisometry is presented. This technique allows for reconstruction of the MPFL as close as possible to its nature with a flat and wide patellar attachment and a gradual transition of maximal tension across the graft fibers, depending on the knee flexion angle. Precise intraoperative control of predefined graft angular anisometry allows for achievement of the desired amount of tension at specific flexion angles. This mitigates the risk of under- or overtensioning and subsequently ensures proper medial-lateral mobility of the patella.

The medial patellofemoral ligament (MPFL) is the primary medial stabilizer of the patella during the first 30**°** of knee flexion and is ruptured in as many as 98% of lateral patellar dislocation cases.^1^^,^^2^ MPFL reconstruction (MPFLR) is an important procedure in the treatment of lateral patellar instability and may be performed alone or in combination with other procedures.^3^, ^4^, ^5^ Various MPFLR techniques were proposed, commonly using hamstring tendon auto- or allografts.^6^ However, the tubular structure of these grafts poses limitations in accurately mimicking the flat, sail-like anatomy of the native MPFL, with distinct tension on the proximal and distal parts of the ligament.^2^^,^^7^, ^8^, ^9^, ^10^ Furthermore, over- or undertensioning of the graft due to femoral tunnel misplacement remains a significant risk. This is not accurately solved either by the intraoperative fluoroscopic landmark identification, due to rotational errors of the true lateral radiographs or even due to unreliability of the radiographic reference points themselves,^11^, ^12^, ^13^, ^14^, ^15^, ^16^, ^17^ or by palpation of the anatomic footprints, due to differences in distances from the femoral MPFL attachment to the anatomic reference points between patients.^2^^,^^18^

Therefore, we propose an alternative MPFLR technique with fascia lata allograft that aims to restore the natural shape of the MPFL as close as possible, with flat and wide patellar attachment and the gradual transition of maximal tension across the graft fibers, depending on the knee flexion angle. Precise intraoperative control of predefined graft angular anisometry allows for the achievement of the desired amount of tension at specific flexion angles. This mitigates the risk of under- or overtensioning and subsequently ensures proper medial-lateral mobility of the patella.

## Surgical Technique

### Graft Preparation

**Fig 1:**
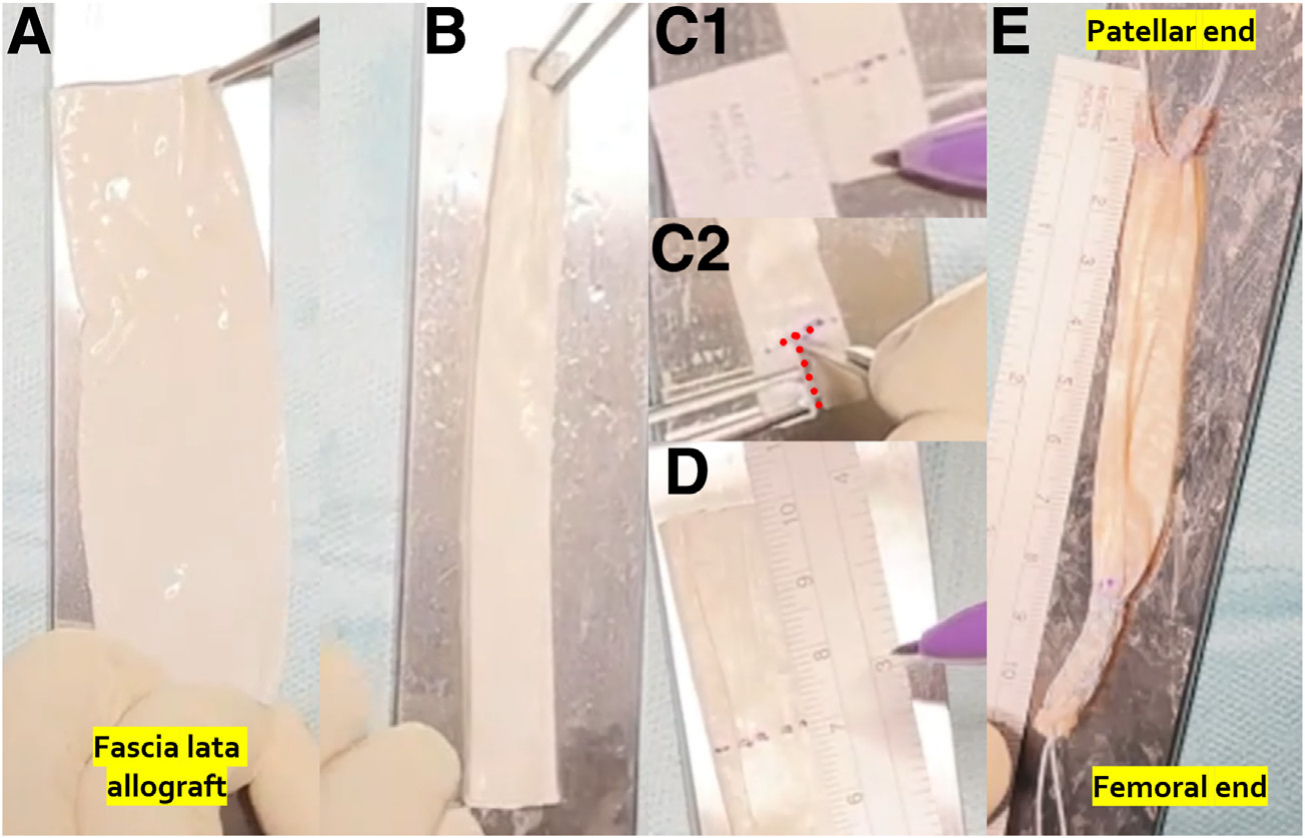
(A) A fascia lata allograft—note that it is wide and flat—similar to the native medial patellofemoral ligament. (B) Fascia lata allograft folded in 3 along the long axis. (C) “T-shape” 12 mm from the patellar end of the graft is marked (C1) and cut to about the middle of the “arms” width (C2, red dots). (D) A total of 20 to 30 mm from the femoral end of the graft is marked, leaving 65 to 75 mm of the free part of the graft. (E) Both ends of the graft are sutured with MaxBraid suture (ZimmerBiomet), using a standard Krackow stitch.

A fascia lata allograft of a minimum 10 cm long and 3 cm width is required (Fig 1A). It is then folded into 3 along the long axis (Fig 1B), marked and cut in a “T-shape” 12 mm from the patellar end of the graft (Fig 1C), and marked 20 to 30 mm from the femoral end of the graft, so that 65 to 75 mm of the free part of the graft is left (Fig 1D). Both ends of the graft are sutured with a MaxBraid suture (ZimmerBiomet) using a standard Krackow stitch (Fig 1E). The patellar “arms” of the graft cannot be thicker than 3.2 mm in diameter, while the femoral “arm” is usually between 5 and 6 mm in diameter.

### Access to the Patella

**Fig 2:**
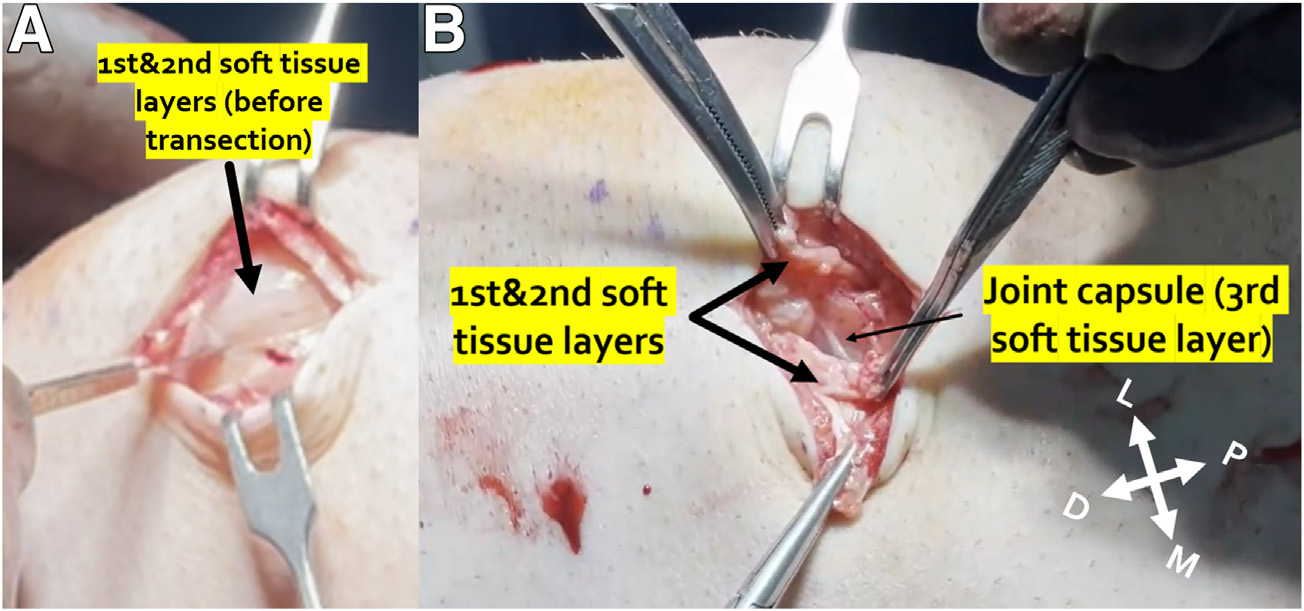
Right knee. (A) A 3- to 4-cm skin incision. (B) First and second layers of the medial soft tissues are transected and elevated, and the joint capsule is visible in the bottom of the incision. (D, distal; L, lateral; M, medial; P, proximal.)

A 3- to 4-cm-long skin incision is performed along the medial border of the patella (Fig 2A). The first and second layers of the medial soft tissues are transected, taking care to leave the third layer, the joint capsule, intact (Fig 2B). Natively, the MPFL is located relatively deep in the medial soft tissues, with only the joint capsule deep to it.^19^, ^20^, ^21^ We reconstruct MPFL in the natural soft tissue path between the second and third soft tissue layers so that the graft lays on the joint capsule.

### Patellar Tunnels

**Fig 3:**
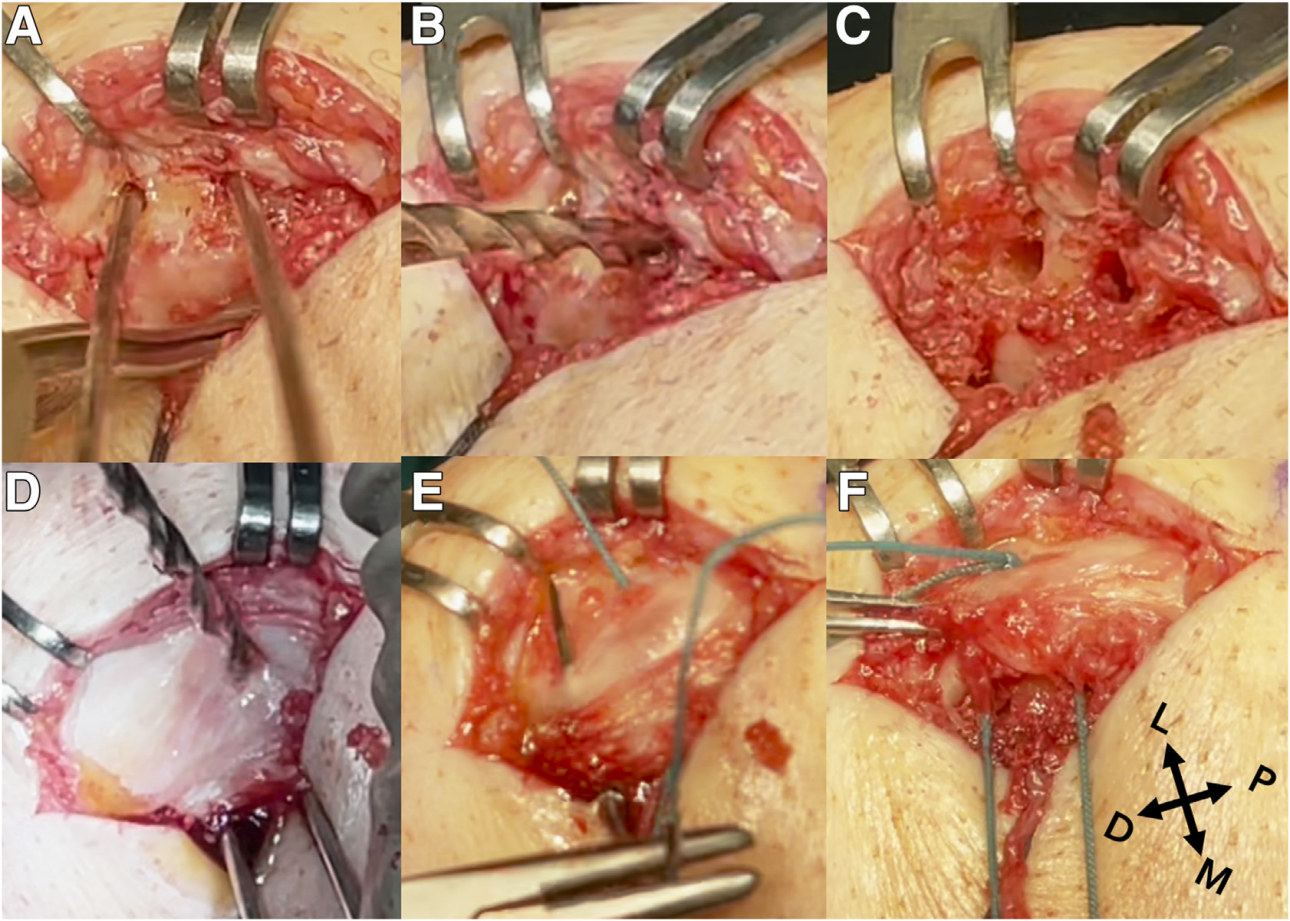
Right knee. (A) Two K-wires are used to mark the location of the patellar tunnels. (B, C) The 3.2-mm-diameter tunnels are drilled to the depth of 12 to 14 mm, taking care to leave at least a 3- to 4-mm bone bridge from the anterior bony surface of patella. (D) Perpendicular tunnels are drilled on the anterior surface of patella 10 to 12 mm from its medial margin until the drill touches the K-wires reinserted in the tunnels. (E) Passing sutures (Ethibond; Johnson & Johnson) are passed through the patellar tunnels. (F) A knot is tied on the anterior patellar surface. (D, distal; L, lateral; M, medial; P, proximal.)

Two K-wires are used to mark the location of the patellar tunnels. The distal tunnel is located in the middle of the proximal-distal length of the patella, which is usually its most prominent part. The second one is located approximately 1 cm proximally from the distal tunnel (Fig 3A). The 3.2-mm diameter tunnels are drilled to a depth of 12 to 14 mm, taking care to leave at least a 3- to 4-mm bone bridge from the anterior bony surface of the patella (Fig 3 B and C). K-wires are reinserted into the prepared tunnels, and perpendicular tunnels are drilled on the anterior surface of the patella, 10 to 12 mm from its medial margin, until the drill touches the K-wires in the tunnels (Fig 3D). Passing sutures (Ethibond; Johnson & Johnson) are passed through the patellar tunnels, and a knot is tied on the anterior patellar surface (Fig 3 E and F).

### Search for Femoral Tunnel Location

**Fig 4:**
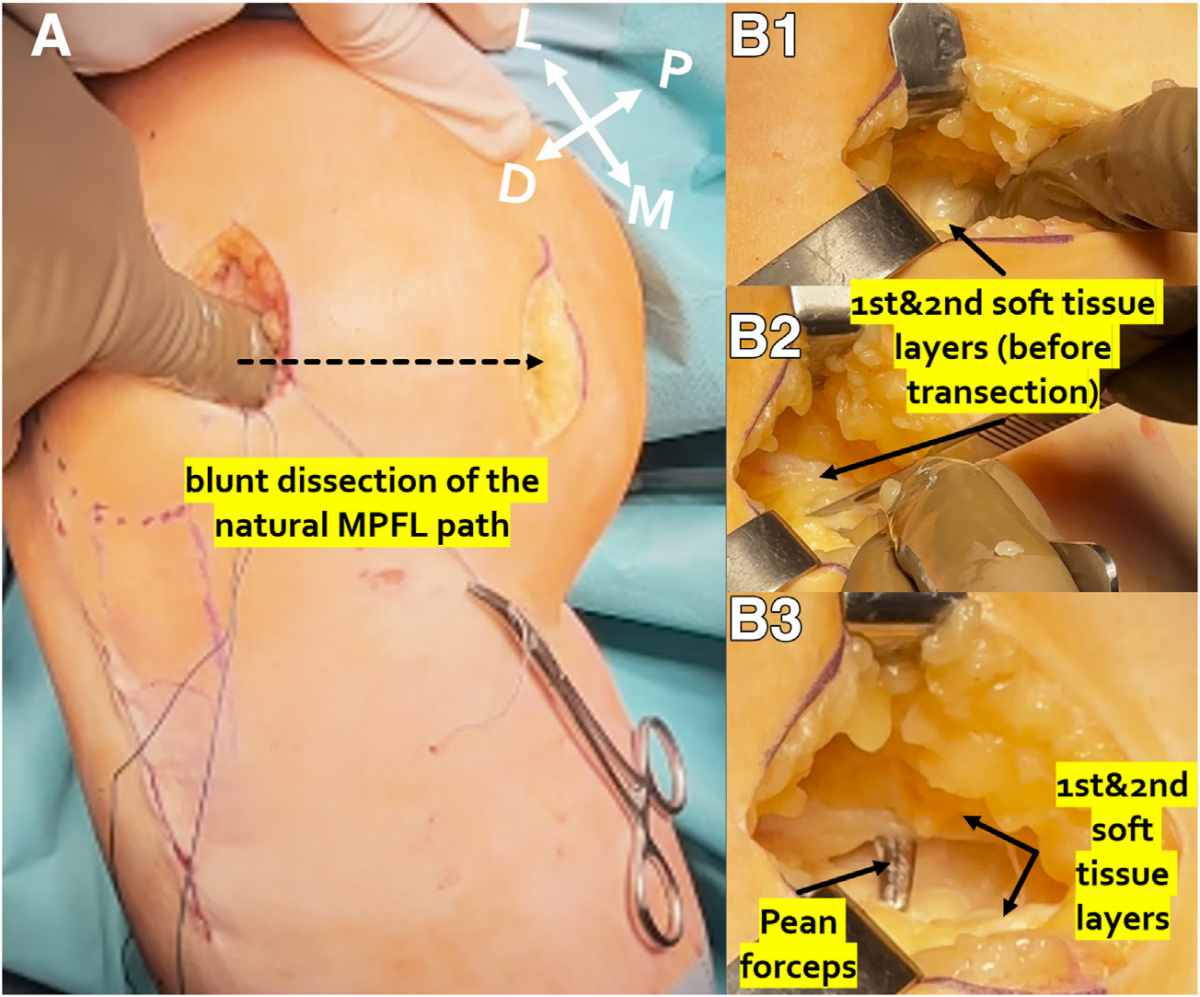
Right knee. (A) Blunt dissection of the natural MPFL path just above the third soft tissue layer with the finger. (B) Pean forceps introduced through the found path are used to elevate the first and second tissue layers and the tissues are transected. (D, distal; L, lateral; M, medial; MPFL, medial patellofemoral ligament; P, proximal.)

**Fig 5:**
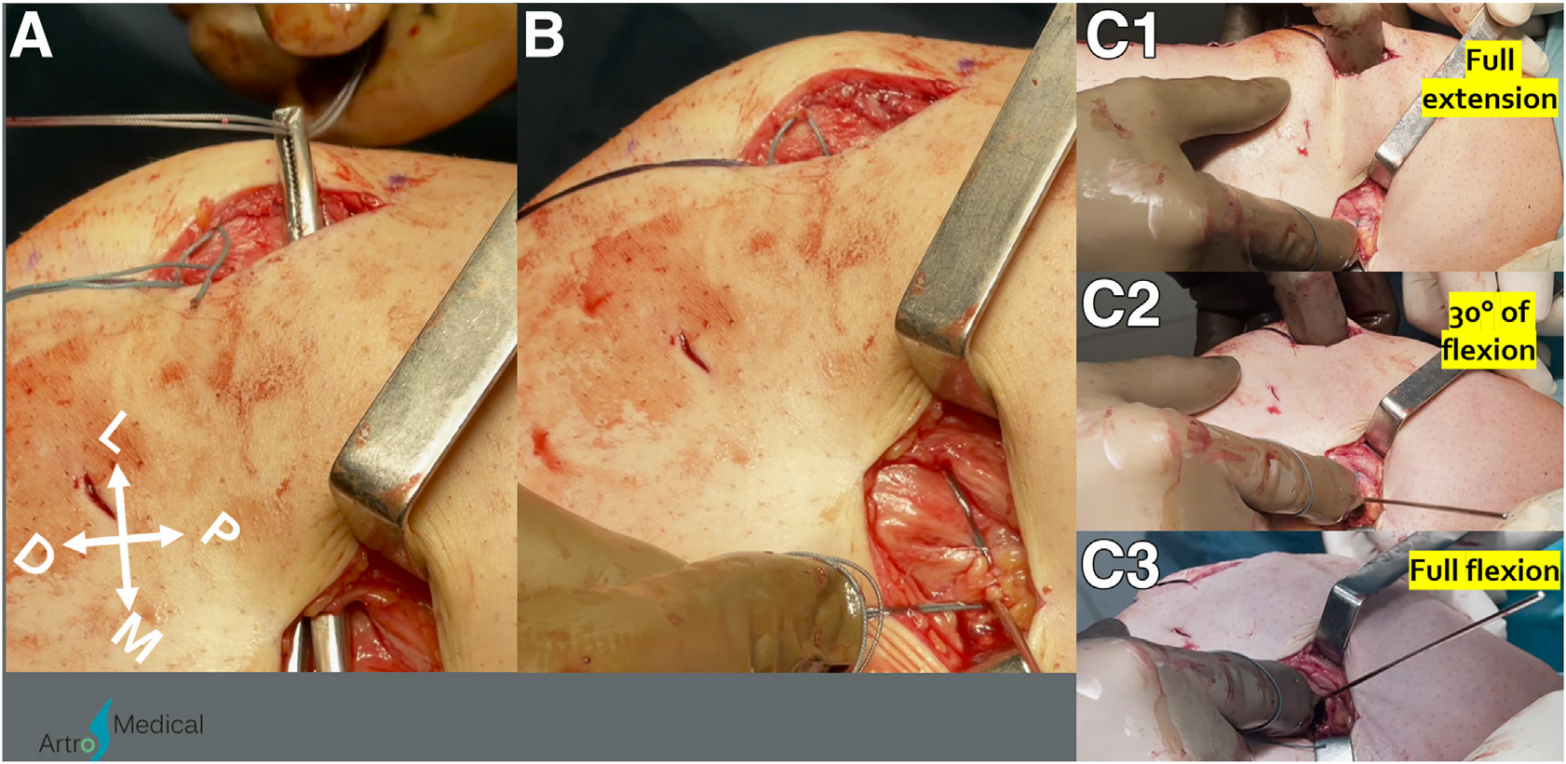
Right knee. (A) The patellar sutures are drawn through the medial patellofemoral ligament path found in the previous steps. (B) The K-wire is drilled in the expected location and sutures are wrapped around the K-wire. (C) The graft tension and patellar mobility in specific knee flexion angles from full extension to full flexion are assessed. (D, distal; L, lateral; M, medial; P, proximal.)

A second skin incision is performed in the area of the medial femoral epicondyle (MFE), adductor tubercle (AT), and gastrocnemius tubercle, as confirmed by the blunt dissection of the natural MPFL path just above the third soft tissue layer with a finger (Fig 4A). Then, pean forceps are introduced through the soft tissue tunnel path, and the first and second tissue layers are elevated with a tip of the forceps and transected (Fig 4B). After the MFE and AT are identified, a search of the femoral attachment is begun approximately 10 mm proximally, 9 mm dorsally from the MFE and 4 mm distally, 2 mm ventrally from the AT.^22^ The patellar sutures are passed through the MPFL path found in the previous steps (Fig 5A). The K-wire is drilled in the expected location and sutures are wrapped around the K-wire (Fig 5B). Then, the graft tension and patellar mobility in specific flexion angles from full extension to full knee flexion are assessed (Fig 5C). The femoral attachment localization will greatly influence the graft angular anisometry, enabling or blocking the desired amount of tension at specific flexion angles. The goal of a MPFLR is not to reconstruct the “string” in between the femur and patella but to restore proper medial-lateral mobility of the patella by reconstructing the ligament, which is taut in 20**°** to 30**°** of flexion and a bit naturally lax in full extension and deep flexion.^9^^,^^23^ If the angular anisometry is different, it is recommended to keep searching for the correct femoral tunnel localization.

### Femoral Tunnel and Fixation

**Fig 6:**
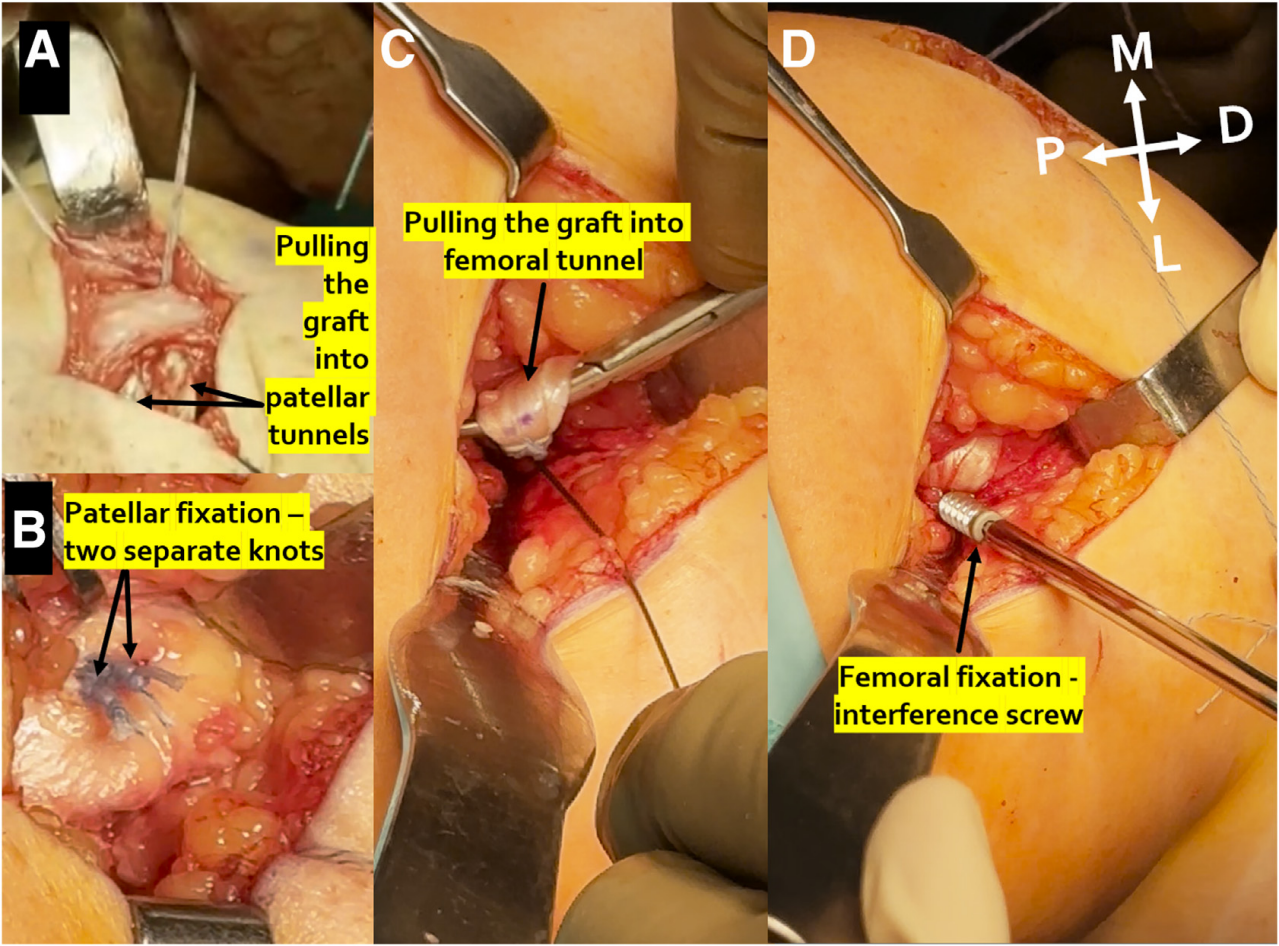
Left knee. (A) The graft is pulled into the patellar tunnels. (B) Two separate knots on the anterior surface of patella are used to fix the graft. (C) The graft is pulled into the femoral tunnel, taking into account not to twist it. (D) Fixation with an interference screw, with the knee flexed to about 30°—at the location of the largest tension on the medial patellofemoral ligament graft. (D, distal; L, lateral; M, medial; P, proximal.)

Femoral tunnel size corresponding to the diameter of the femoral end of the graft is drilled to a depth of 3 to 3.5 cm, which is 1 to 1.5 cm more than the measured femoral socket portion of the graft, to have the possibility of regulating tension. The suture passer wire is used to pass the sutures. The graft is pulled into the patellar tunnels (Fig 6A) and secured by means of 2 separate knots on the anterior surface of the patella (Fig 6B). Then, the graft is pulled into the femoral tunnel, taking into account not to twist it (Fig 6C) and fixed with an interference screw (Fig 6D), with the knee flexed to about 30**°—**at the location of the largest tension on the MPFL graft. Then, a closure of the medial soft tissue layers is performed. Subsequent sutures are performed with delayed knot tying in order to not close the visibility and not to suture the MPFL graft to the second layer of the soft tissues (Video 1). Tips and tricks are summarized in Table 1.

**Table 1:** Tips and Tricks

If MPFLR is combined with other procedures, such as tibial tuberosity osteotomy, derotational osteotomy, or trochleoplasty, MPFLR should be performed as a last step.
Proper identification of medial soft tissue layers is crucial.
Patellar “arms” of the graft cannot be thicker than 3.2 mm in diameter—meticulous graft preparation is necessary.
It is crucial to leave at least a 3- to 4-mm bone bridge on the anterior bony surface of the patella during patellar tunnel preparation.
Both patellar “arms” of the graft have to be tightly pulled into the patella.
Avoid twisting the graft.
Interference screw is a preferred fixation method rather than an endobutton due to possible irreversible overconstriction if a tensioning mistake occurred.
The goal of MPFLR is not to reconstruct the “string” in between the femur and patella but to restore the proper medial-lateral mobility of the patella by reconstructing the ligament, which is taut when necessary (in 20**°**-30**°** of flexion) and lax when necessary (a little bit of natural laxity in full extension and laxity in deep flexion).
Take great care not to suture the MPFL graft to the second layer of soft tissues during closure of medial soft tissue layers.

MPFL, medial patellofemoral ligament; MPFLR, medial patellofemoral ligament reconstruction.

### Rehabilitation

Rehabilitation includes 6 weeks of straight knee bracing and crutches for ambulation, as well as 5 minutes of gravitational hyperextension at maximal flexion every 2 hours starting the first postoperative day. After 2 weeks, the patient starts guided physiotherapy twice a week. Patellar mobilizations are emphasized in the early stages, while strengthening and functional improvement are emphasized after 6 weeks.

## Discussion

Determination of the native MPFL angular anisometry remains a challenge^9^ and may be influenced by anatomic differences between patients and by the fibers of the quadriceps muscle intermeshed with the MPFL.^2^^,^^9^^,^^10^^,^^18^^,^^23^^,^^24^ The goal of this MPFLR technique is to reconstruct the ligament, which is taut in 20**°** to 30**°** of flexion and therefore prevents lateral patellar instability.^1^^,^^2^ However, a little bit of “natural” laxity in full extension and in deep flexion is necessary as well, to avoid the risk of excessive cartilage compression (in case the graft does not stretch) or recurrent patellar instability (in case the graft does stretch gradually).^9^^,^^23^ In the proposed technique, precise intraoperative control of graft angular anisometry allows for achievement of the desired amount of tension in knee flexion angles, mitigating the abovementioned risks of under- or overtensioning. In addition, a flat and wide patellar attachment allows for gradual transition of maximal tension across the graft fibers, depending on the knee flexion angle. Importantly, this technique allows for finding the optimal angular anisometry even in cases with complicated distal femur and patella morphology. This is because the surgeon can examine the graft tension with their own hand, which is not possible in techniques in which only anatomic and/or radiologic footprints are used, without intraoperative anisometry assessment being performed.^2^^,^^11^, ^12^, ^13^, ^14^, ^15^^,^^18^ Advantages and disadvantages of the technique are summarized in the Table 2.

**Table 2:** Advantages and Disadvantages of the Technique

Advantages	Disadvantages
Restoration of a flat, wide patellar attachment with distinct tension of the proximal and distal parts of the graft.	Necessity of a fascia lata allograft with associated cost.
Intraoperative control of graft isometry/tension and of subsequent patellar medial-lateral mobility mitigates the risk of under- or overtensioning.	Longer femoral incision—necessary for intraoperative search of femoral tunnel localization resulting in proper graft angular anisometry.
Small diameter of patellar tunnels, diminishing the risk of patellar fracture.	More tedious graft preparation than hamstrings.
No hardware is used in the patella, allowing for bone-tendon healing at the whole diameter of the tunnels.	
Radiologic assessment is not necessary, lessening the radiation exposure of the personnel and the patient.	
Gradual transition of maximal tension across the graft fibers, depending on the knee flexion angle.	

## Disclosures

All authors (M.M., M.B., K.B., P.A.P., R.L.F., K.M.) declare that they have no known competing financial interests or personal relationships that could have appeared to influence the work reported in this paper.

## Supplementary Data

Video 1 Left knee. Fascia lata allograft of a minimum 10-cm length and 3-cm width is required. It is then folded in 3 along the long axis, marked and cut in a “T-shape” 12 mm from the patellar end of the graft, and marked 20 to 30 mm from the femoral end of the graft and both patellar and femoral arms are sutured. A 3- to 4-cm-long skin incision is performed along the medial border of the patella. The first and second layers of the medial soft tissues are transected, taking care to leave the third layer—joint capsule—intact. Natively, the medial patellofemoral ligament (MPFL) is located relatively deep in the medial soft tissues, with only the joint capsule beneath it. We reconstruct the MPFL in the natural soft tissue path between the second and third soft tissue layers so that the graft lays on the joint capsule. Two K-wires are used to mark the location of patellar tunnels. Two 3.2-mm-diameter tunnels are drilled to the depth of 12 to 14 mm, leaving at least a 3- to 4-mm bone bridge from the anterior bony surface of patella. Perpendicular tunnels are drilled 10 to 12 mm from the medial margin of the patella until the drill touches the K-wires in the tunnels. Passing sutures (Ethibond; Johnson & Johnson) are passed through the patellar tunnels and a knot is tied on the anterior patellar surface. Blunt dissection of the natural MPFL path just above the joint capsule is performed. The search for the femoral attachment is begun approximately 10 mm proximally, 9 mm dorsally from the medial femoral epicondyle and 4 mm distally, 2 mm ventrally from the adductor tubercle.^22^ The patellar sutures are drawn through the MPFL path. The K-wire is drilled in the expected location of the femoral tunnel and sutures are wrapped around it. We assess the graft tension and patellar mobility in specific flexion angles. This is the crucial part of the procedure—more details are included in the article. The femoral tunnel is drilled to a depth of 3 to 3.5 cm, which is 1 to 1.5 cm more than the measured femoral socket portion of the graft, to have the possibility of regulating tension. The graft is pulled into the patellar tunnels and secured by 2 separate knots on the anterior surface of the patella. Then, the graft is pulled into the femoral tunnel, taking into account not to twist it, and it is fixed with the interference screw with the knee flexed to about 30**°—**at the location of the biggest tension that occurs on the MPFL graft. Then, a closure of the medial soft tissue layers is performed. Subsequent sutures are performed with delayed knot tying in order to not close the visibility and not suture the MPFL graft to the second soft tissue layer.
